# Sirolimus Therapy and Follow-up in a Patient with Severe Congenital Hyperinsulinism Following Subtotal Pancreatectomy

**DOI:** 10.4274/jcrpe.galenos.2020.2020.0033

**Published:** 2021-02-26

**Authors:** Qiong Chen, Yongxing Chen, Xiaohong Wang, Haihua Yang, Yingxian Zhang, Xiaojing Liu, Yun Yan, Haiyan Wei

**Affiliations:** 1 Henan Children’s Hospital (Children’s hospital affiliated to Zhengzhou University), Department of Endocrinology and Metabolism, Genetics, Zhengzhou, China; 2 University of Missouri-Kansas City, Children’s Mercy Hospital, Department of Endocrinology and Diabetes, Missouri, USA

**Keywords:** Congenital hyperinsulinism, hypoglycemia, mTOR, sirolimus, ABCC8

## Abstract

Congenital hyperinsulinism (CHI) is the most common cause of severe, persistent hypoglycemia in neonates and infants. If the patient does not respond to medical treatment the currently available treatment is subtotal pancreatectomy, but some patients still experience severe hypoglycemia after surgery. Sirolimus, a mammalian target of rapamycin inhibitor has recently been reported to be effective in the treatment of insulinoma and CHI patients. Here we report a patient with CHI who had prolonged hypoglycemia after subtotal pancreatectomy. The patient had a heterozygous mutation in ABCC8 but was unresponsive to an optimal dose of diazoxide (15 mg/ kg/day) and octreotide (30 μg/kg/day). The patient subsequently had subtotal pancreatectomy but severe and persistent hypoglycemia continued post-operatively. Sirolimus was commenced. There was a remarkable improvement in glycemic control without major adverse events, although he required a small dose of octreotide to maintain euglycemia. Sirolimus therapy was discontinued when the patient was 15 months old. At the time of this report, at an age of three years and eight months, the patient continues to maintain good glycemic control. This report suggests that sirolimus may be an effective treatment option in patients with CHI resistant to established medical therapy or failure of subtotal pancreatectomy. However, the long-term safety requires study in larger groups of very young patients.

**What is already known on this topic?**

Congenital hyperinsulinism (CHI) is the most common cause of persistent hypoglycemia in neonates and infants. Sirolimus may be an effective treatment option in patients with CHI resistant to traditional medical therapy or failure of subtotal pancreatectomy, but experience is limited.

**What this study adds?**

This article further revealed the safety and efficacy of sirolimus in the very young patient with CHI, which can make us better understand the new treatment. The patient has a heterozygous ABCC8 mutation. It’s also the first Chinese CHI patient with heterozygous ABCC8 mutation who used sirolimus.

## Introduction

Congenital hyperinsulinism (CHI), the major cause of persistent hypoglycemia in neonates and infants, is characterized by inappropriate insulin secretion from pancreatic beta cells in the presence of low blood glucose levels (1). Prompt and early management of these patients is very important for neurological prognosis (1,2). The incidence of CHI in the general population is estimated at 1/30,000-1/50,000 live births (3,4). Two major histologic subtypes have been described: diffuse (60-70% of patients) and focal (30-40% of patients) (5). Mutations in *ABCC8* and *KCNJ11* cause severe CHI that is unresponsive to medical treatment with diazoxide and octreotide (1). The current treatment for patients is a subtotal pancreatectomy (5,6). However, despite surgery, 40-59% of operated patients continue to experience severe and persistent hypoglycemia for months, or even years (7), and nearly 100% will develop diabetes mellitus within 11 years of surgery (8). Therefore, medical therapeutic alternatives should be considered with the aim of reducing insulin secretion and thereby preventing neurologic consequences. Constitutive activation of the mammalian target of rapamycin (mTOR) pathway has been postulated as a mechanism for hyperinsulinism and β-cell hyperplasia in diffuse CHI (9). Recent advances have shown the effectiveness of sirolimus, an mTOR inhibitor, in infants with severe diffuse CHI that had been unresponsive to medical therapy (10,11,12,13), one of whom had undergone subtotal pancreatectomy (10). During follow-up, no major adverse events was observed in the patients. We report a patient with CHI who failed to become euglycemic after pancreatectomy. The patient was successfully treated with sirolimus without further surgical intervention.

## Case Report

**Figure 1:**
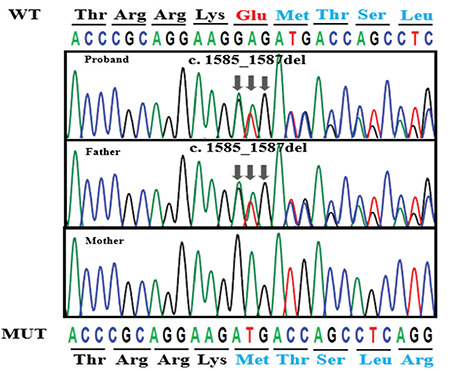
Sanger sequencing of ABCC8 gene in the proband and his parents: the arrows showed the mutation site of the *ABCC8* gene

**Figure 2:**
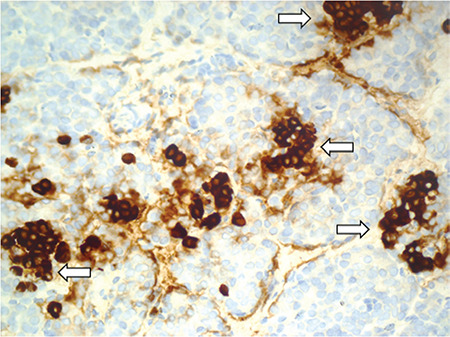
Histopathological result confirmed diffuse hyperplasia of the islet cells. The arrows showed hypersecretion of islet cells in islets

The patient, a male infant, was born by cesarean at the 39^th^ week of gestation to nonconsanguineous Chinese parents after an uneventful pregnancy. Birth weight was 3600 g. On the first day of his life, he was found to have severe hypoglycemia when he developed lethargy and seizures. He required high intravenous glucose infusion rate (GIR) (13 mg/kg/minute) to maintain normal blood glucose level. As he had persistent and severe hypoglycemia, he was transferred to our hospital for further management on postnatal day (PD) 13. The following results were obtained during an episode of hypoglycemia: glucose, 2.3 mmol/L; concomitant serum insulin, 13.52 (normal: 4.03-23.46) µIU/mL; C-peptide 4.25 (normal: 0.3-3.73) ng/mL; beta-hydroxybutyrate  < 0.1 mmol/L. He had normal thyroid-stimulating hormone and free T4 levels. Metabolic screening profiles in plasma and urine were non-specific. Genetic analysis subsequently confirmed a novel mutation, c.1585_1587del, in exon 10 of the *ABCC8* gene (Figure 1), which resulted in the deletion of glutamic acid at position 529 (p.del529E) of *ABCC8* protein and produced *ABCC8* protein with a shorter topological domain (www.ncbi.nih.gov/orffinder and www.uniprot.org). The mutations in the topological domain affected the function of the *ABCC8* gene (14). According to American College of Medical Genetics criteria, the mutation is of uncertain significance and should be further studied (15). His father has the same mutation, but the phenotype is normal. Magnetic resonance imaging of the patient’s brain showed bilateral abnormalities of the parietal white matter. Subsequently, maximal GIR was 16 mg/kg/min, administered parenterally via a central venous catheter. Diazoxide therapy was commenced on PD 14 and was gradually increased to an optimal dose of 15 mg/kg/day but with no response. On PD 20 Nifedipine was added to the therapeutic regimen, but it was discontinued after a week due to lack of response. Subcutaneous octreotide was initiated on PD 29. The octreotide dose was increased to a maximum dose (30 µg/kg/day), but resulted in only a 20% reduction in total glucose requirement. On PD 55, a subtotal pancreatectomy was performed at the Children’s Hospital of Fudan University, Shanghai, China. Histopathological results confirmed diffuse hyperplasia of the islet cells (Figure 2). Subcutaneous octreotide was discontinued after the surgery, but the minimum GIR remained 10 mg/kg/min. Octreotide subcutaneous injection was resumed with the dose of 30 µg/kg/day. Over the next few weeks, there was no reduction in his glucose requirement. The total volume of nasogastric and parenteral fluids reached 190 mL/kg/d and it was also very difficult to establish a central venous line.

In view of the multiple medical problems, further surgery was being contemplated. After reviewing the risks and beneﬁts, Sirolimus was considered as an alternative treatment option. Sirolimus treatment was begun at 4.5 months of age at a dose of 0.5 mg/m^2^/day. The dose was gradually increased, with the goal of reaching a serum trough level of 5-15 ng/dL. The serum trough level of sirolimus was measured every 5-7 days. After 10 days of treatment with sirolimus, intravenous glucose infusion and subcutaneous octreotide were gradually tapered. Four weeks following initiation of sirolimus, stable blood glucose homeostasis was achieved without intravenous glucose infusion, and the octreotide dose was reduced from 30 µg/kg/day to 15 µg/kg/day. The patient was able to tolerate fasting for four hours while maintaining a blood glucose level >60 mg/dL prior to discharge as recorded by continuous glucose monitoring. The patient was followed regularly for assessment of glycemic control and measurement of serum sirolimus levels. Sirolimus was discontinued at 15 months of age. The maximum dose of sirolimus used was 3.2 mg/m^2^/day.

The patient had good glycemic control after cessation of sirolimus. However, at times of poor appetite, there was still a requirement for low dose octreotide (2 µg/kg/day) to control blood glucose. Complete blood count, serum lipid profile, and renal and liver function have been monitored regularly and no significant side effects were observed, except for mildly elevated triglycerides at two years and three months old. At the time of writing the patient was three years and six months old. At the last visit, the patient was able to tolerate fasting for six hours according to continuous glucose monitoring. The blood glucose was 95 mg/dL and the insulin was 4.9 µIU/mL at the end of the six hours fasting.

## Discussion

The management of diffuse CHI that is unresponsive to diazoxide poses a major therapeutic challenge. While subtotal pancreatectomy remains the procedure of choice following failure of medical therapy, the surgery is not completely curative and may still be associated with unsatisfactory glycemic control. Fluorine-18-dihydrophenylalanine positron emission tomography was not performed in our patient before surgery, since it was not available in children in China at the time, but based on the increased number of islets and enlarged volume of partial regional islets reported histopathologically, as well as the recurrent severe hypoglycemia after subtotal pancreatectomy, the patient likely had a diffuse CHI. The patient has a heterozygous mutation in ABCC8. Definitely, most dominant acting monoallelic potassium channel ATP gene mutations cause mild diazoxide responsive CHI. However, Saint-Martin et al (16) reported that some dominant *ABCC8* mutations are responsible for a subset of diffuse, diazoxide-unresponsive forms of CHI. The mechanism in these cases is unclear and needs further study. After pancreatectomy, the total amount of nasogastric and parenteral fluids had reached maximum, and it was also very difficult to establish a central venous line. Therefore, there was a need for an alternative treatment, minimizing the requirement for repeat pancreatectomy, and the burden of demanding medical and nutritional intervention in our patient.

Sirolimus has been reported as a treatment option for unresponsive CHI (10,11,12,13). No major adverse reactions were observed during follow-up period in these case reports, though a recent study in two large centers showed that mTOR inhibition achieved euglycemia, fasting tolerance and reduced medical therapy in only 30% of patients and more adverse events were observed (17). mTOR is a serine and threonine protein kinase that integrates signals from mitogens and nutrients, glucose and amino acids, to regulate cellular growth and proliferation (18). The mechanism of mTOR inhibitors in CHI has not been fully delineated. Hyperplasia of β-cells has been proposed to be involved in the trans-differentiation of mature acinar and ductal elements of exocrine pancreas into insulin-secreting cells, which is possibly mediated by the constitutive activation of the mTOR pathway (19). mTOR inhibition may also affect the number of insulin receptors that are present in pancreatic β-cells, which would reduce insulin production (20). Sirolimus has been used *in vitro* to induce fulminant diabetes by promoting insulin resistance and reducing β-cell mass through apoptosis induction (21,22). Furthermore, long-term management with sirolimus was found to cause glucose intolerance by up-regulating hepatic gluconeogenesis (23). It is postulated that the mechanism of mTOR inhibition is also reduced during islet cell proliferation (11,12). This was recently confirmed by genomic datasets implicating the insulin-like growth factor 1/mTOR/Akt pathway in the pathophysiology of CHI (9). Another study has shown that mTOR pathways are not downregulated in keeping with non-responsiveness to sirolimus and the observation that proliferation remains high after treatment with sirolimus (17).

The reported adverse effects of sirolimus treatment include stomatitis, fatigue, immunosuppression, increased risk of infections, renal function abnormalities, hyperlipidemia, and pneumonitis (22,24), which are reversible with dose reduction. Mild elevation of triglycerides was observed in our patient. Sirolimus appears to be well tolerated in children post renal transplant, even when initiated at higher doses (6 mg/m^2^/day) and thereafter adjusted to achieve target trough levels in the range of 10-20 ng/mL (25). These studies suggest a reasonable safety profile for sirolimus, but the long-term safety remains unknown in younger children, particularly in neonates.

## Conclusion

In conclusion, sirolimus was a well-tolerated treatment in our patient with CHI who otherwise would have required second surgery, and no major adverse events were observed during the period of 10 months of treatment. Sirolimus may be a feasible option for selected CHI patients with no contraindication, either before surgery or as an adjunctive therapy, although the mechanism and long-term adverse effects of such treatment require further study.
